# Sodium rubidium hydrogen citrate, NaRbHC_6_H_5_O_7_, and sodium caesium hydrogen citrate, NaCsHC_6_H_5_O_7_: crystal structures and DFT comparisons

**DOI:** 10.1107/S205698901900063X

**Published:** 2019-01-18

**Authors:** Andrew J. Cigler, James A. Kaduk

**Affiliations:** a Department of Chemistry, North Central College, 131 S. Loomis St., Naperville IL 60540, USA

**Keywords:** powder diffraction, density functional theory, citrate, sodium, rubidium, caesium, crystal structure

## Abstract

The crystal structure of sodium rubidium hydrogen citrate, NaRbHC_6_H_5_O_7_ or [NaRb(C_6_H_6_O_7_)]_*n*_, has been solved and refined using laboratory powder X-ray diffraction data, and optimized using density functional techniques. This compound is isostructural to NaKHC_6_H_5_O_7_. The Na atom is six-coordinate, with a bond-valence sum of 1.16. The Rb atom is eight-coordinate, with a bond-valence sum of 1.17. The distorted [NaO_6_] octa­hedra share edges to form chains along the *a*-axis direction. The irregular [RbO_8_] coordination polyhedra share edges with the [NaO_6_] octa­hedra on either side of the chain, and share corners with other Rb atoms, resulting in triple chains along the *a*-axis direction. The most prominent feature of the structure is the chain along [111] of very short, very strong hydrogen bonds; the O⋯O distances are 2.426 and 2.398 Å. The Mulliken overlap populations in these hydrogen bonds are 0.140 and 0.143 electrons, which correspond to hydrogen-bond energies of about 20.3 kcal mol^−1^. The crystal structure of sodium caesium hydrogen citrate, NaCsHC_6_H_5_O_7_ or [NaCs(C_6_H_6_O_7_)]_*n*_, has also been solved and refined using laboratory powder X-ray diffraction data, and optimized using density functional techniques. The Na atom is six-coordinate, with a bond-valence sum of 1.15. The Cs atom is eight-coordinate, with a bond-valence sum of 0.97. The distorted trigonal–prismatic [NaO_6_] coordination polyhedra share edges to form zigzag chains along the *b-*axis direction. The irregular [CsO_8_] coordination polyhedra share edges with the [NaO_6_] polyhedra to form layers parallel to the (101) plane, unlike the isolated chains in NaKHC_6_H_5_O_7_ and NaRbHC_6_H_5_O_7_. A prominent feature of the structure is the chain along [100] of very short, very strong O—H⋯O hydrogen bonds; the refined O⋯O distances are 2.398 and 2.159 Å, and the optimized distances are 2.398 and 2.347 Å. The Mulliken overlap populations in these hydrogen bonds are 0.143 and 0.133 electrons, which correspond to hydrogen-bond energies about 20.3 kcal mol^−1^.

## Abstract 2

The crystal structures of sodium rubidium hydrogen citrate and sodium caesium hydrogen citrate have been solved and refined using laboratory X-ray powder diffraction data, and optimized using density functional techniques. In NaRbHC_6_H_5_O_7_, the Na and Rb cation coordination spheres form triple chains along the *a*-axis direction, and chains of very strong O—H—O hydrogen bonds run along [111], while in NaCsHC_6_H_5_O_7_ the Na and Cs coordination polyhedra form layers parallel to (101), and there are chains of very short and strong hydrogen bonds along [100].

## Chemical context   

**Figure 1:**
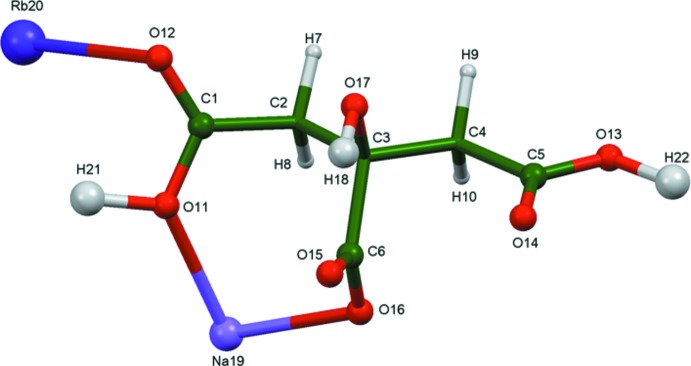
The asymmetric unit of NaRbHC_6_H_5_O_7_, with the atom numbering and 50% probability spheroids.

A systematic study of the crystal structures of Group 1 (alkali metal) citrate salts has been reported in Rammohan & Kaduk (2018 ▸). The study was extended to lithium metal hydrogen citrates in Cigler & Kaduk (2018 ▸). The two title compounds (Figs. 1 ▸ and 2 ▸) are a further extension to citrates that contain more than one alkali metal cation.
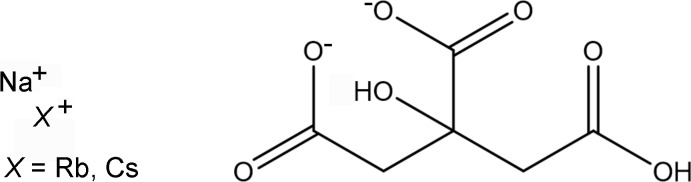


## Structural commentary   

**Figure 3:**
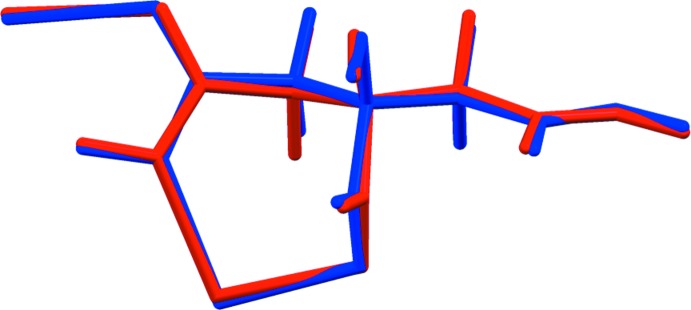
Comparison of the refined and optimized structures of sodium rubidium hydrogen citrate. The refined structure is in red, and the DFT-optimized structure is in blue.

Sodium rubidium hydrogen citrate is isostructural to NaKHC_6_H_5_O_7_ (Rammohan & Kaduk, 2016 ▸). Sodium caesium hydrogen citrate has a related but different structure. The root-mean-square deviations of the non-hydrogen atoms in the refined and optimized structures are 0.116 and 0.105 Å for NaRbHC_6_H_5_O_7_ and NaCsHC_6_H_5_O_7_, respectively. Comparisons of the refined and optimized structures are given in Figs. 3 ▸ and 4 ▸. The excellent agreement between the structures is strong evidence that the experimental structures are correct (van de Streek & Neumann, 2014 ▸). This discussion uses the DFT-optimized structures. All of the citrate bond distances, bond angles, and torsion angles fall within the normal ranges indicated by a *Mercury* Mogul Geometry Check (Macrae *et al.*, 2008 ▸). The citrate anion in both structures occurs in the *trans,trans*-conformation (about C2—C3 and C3—C4), which is one of the two low-energy conformations of an isolated citrate (Rammohan & Kaduk, 2018 ▸). The central carboxyl­ate group and the hy­droxy group occur in the normal planar arrangement.

In the Rb compound, the citrate chelates to Na19 through the terminal carboxyl­ate oxygen O11 and the central carboxyl­ate oxygen O16. The Na^+^ cation is six-coordinate, with a bond-valence sum of 1.16. The Rb^+^ cation is eight-coordinate, with a bond-valence sum of 1.17. Both cations are thus slightly crowded.

In the Cs compound, the citrate triply chelates to Na20 through the terminal carboxyl­ate oxygen O12, the central carboxyl­ate oxygen O15, and the hydroxyl oxygen O17. The Na^+^ cation is six-coordinate, with a bond-valence sum of 1.15. The Cs^+^ cation is eight-coordinate, with a bond-valence sum of 0.97. The Rb—O and Cs—O bonds are ionic, but the Na—O bonds have slight covalent character, according to the Mulliken overlap populations.

The Bravais–Friedel–Donnay–Harker (Bravais, 1866 ▸; Friedel, 1907 ▸; Donnay & Harker, 1937 ▸) method suggests that we might expect a platy morphology for NaRbHC_6_H_5_O_7_, with {001} as the principal faces, and an elongated morphology for NaCsHC_6_H_5_O_7_, with {010} as the long axis. Fourth-order spherical harmonic preferred orientation models were included in the refinements; the texture indices were 1.050 and 1.011, indicating that preferred orientation was slight for the rotated flat-plate specimen of NaRbHC_6_H_5_O_7_, but not significant in this rotated capillary specimen of NaCsHC_6_H_5_O_7_. Examination of the products under an optical microscope indicated that the morphologies were not especially anisotropic.

## Supra­molecular features   

**Figure 5:**
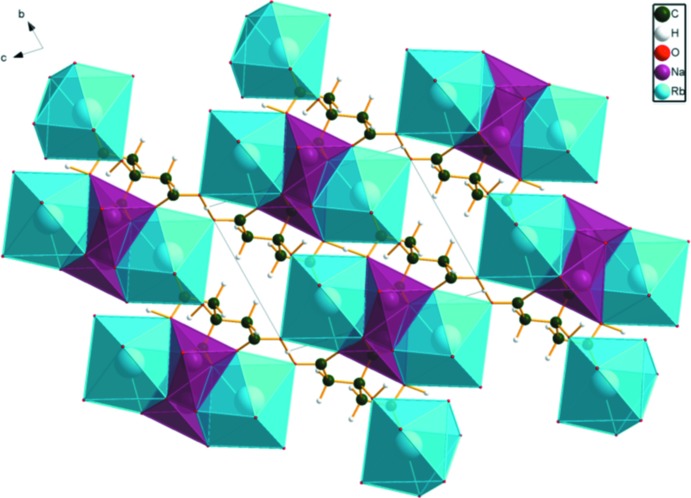
Crystal structure of NaRbHC_6_H_5_O_7_, viewed down the *a* axis.

In the crystal structure of NaRbHC_6_H_5_O_7_ (Fig. 5 ▸), distorted [NaO_6_] octa­hedra share edges to form chains along the *a*-axis direction. The irregular [RbO_8_] coordination polyhedra share edges with the [NaO_6_] octa­hedra on either side of the chain, resulting in triple chains along the *a*-axis direction. The most prominent feature of the structure is the chain along [111] of very short, very strong O—H⋯O hydrogen bonds (Table 1 ▸); the refined O⋯O distances are 2.180 (9) and 2.234 (20) Å, and the optimized distances are 2.426 and 2.398 Å. The Mulliken overlap populations in these hydrogen bonds are 0.140 and 0.143 electrons, which correspond to hydrogen-bond energies about 20.6 kcal mol^−1^, according to the correlation in Rammohan & Kaduk (2018 ▸). H18 forms bifurcated hydrogen bonds: one is intra­molecular to O15, and the other is inter­molecular to O11.

**Figure 6:**
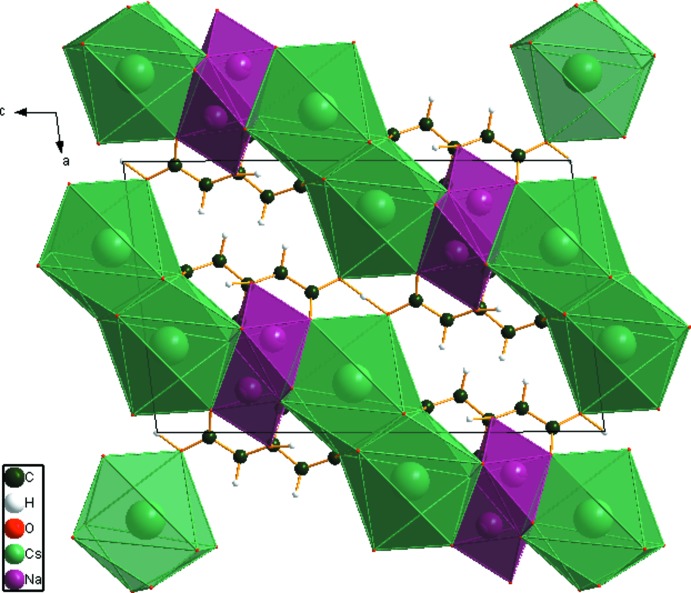
Crystal structure of NaCsHC_6_H_5_O_7_, viewed down the *b* axis.

In the crystal structure of NaCsHC_6_H_5_O_7_ (Fig. 6 ▸), distorted trigonal–prismatic [NaO_6_] share edges to form zigzig chains along the *b*-axis direction. The irregular [CsO_8_] coordination polyhedra share edges with the [NaO_6_] polyhedra to form layers parallel to the (101) plane, unlike the isolated chains in NaKHC_6_H_5_O_7_ and NaRbHC_6_H_5_O_7_. A prominent feature of the structure is the chain along [100] of very short, and very strong O—H⋯O hydrogen bonds (Table 2 ▸); the refined O11⋯O11 and O14⋯O14 distances are 2.398 and 2.159 Å, and the optimized distances are 2.398 and 2.347 Å. The Mulliken overlap populations in these hydrogen bonds are 0.143 and 0.133 electrons, which correspond to hydrogen-bond energies about 20.3 kcal mol^−1^. H18 forms an intra­molecular hydrogen bond to O13, one of the terminal carboxyl­ate oxygen atoms.

## Database survey   

Details of the comprehensive literature search for citrate structures are presented in Rammohan & Kaduk (2018 ▸). After manually locating the peaks in the pattern of NaRbHC_6_H_5_O_7_, the pattern was indexed using *Jade9.8* (MDI, 2017 ▸). A reduced-cell search in the Cambridge Structural Database (CSD Version 5.39, update of November 2018; Groom *et al.*, 2016 ▸) yielded 39 hits, among which was NaKHC_6_H_5_O_7_ (Rammohan & Kaduk, 2016 ▸).

After manually locating the peaks in the pattern of NaCsHC_6_H_5_O_7_, the pattern was indexed on a *C*-centered monoclinic cell using *Jade9.8* (MDI, 2017 ▸). A reduced-cell search in the CSD yielded no hits. The cell was converted to *I*-centered, to yield a β angle closer to 90°.

## Synthesis and crystallization   

Stoichiometric qu­anti­ties of Na_2_CO_3_ and Rb_2_CO_3_ were added to a solution of 10.0 mmol citric acid monohydrate in 10 mL water. After the fizzing subsided, the clear solution was dried in an oven at 403 K to yield the white solid NaRbHC_6_H_5_O_7_.

2.0236 g (10.0 mmol) of H_3_C_6_H_5_O_7_(H_2_O) were dissolved in 10 mL of deionized water. 0.5318 g of Na_2_CO_3_ (1.0 mmol Na, Sigma–Aldrich) and 1.6911 g of Cs_2_CO_3_ (10.0 mmol of Ca, Sigma–Aldrich) were added to the citric acid solution slowly with stirring. The resulting clear colorless solution was evaporated to dryness in a 403 K oven to yield NaCsHC_6_H_5_O_7_.

## Refinement   

**Figure 7:**
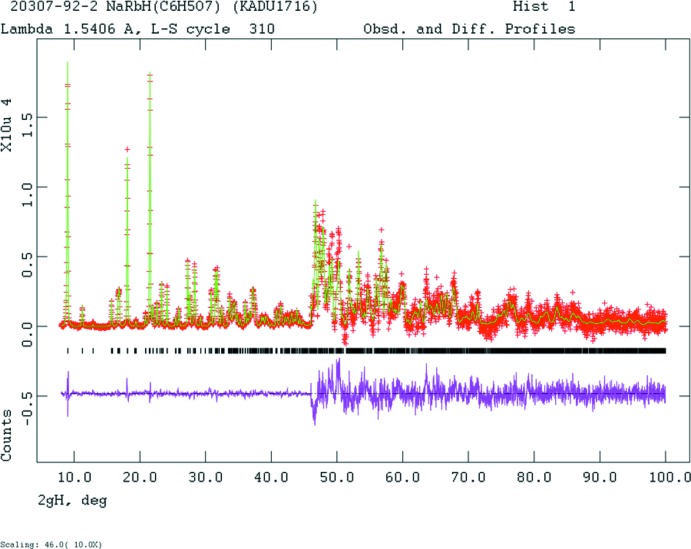
Rietveld plot for NaRbHC_6_H_5_O_7_. The red crosses represent the observed data points, and the green line is the calculated pattern. The magenta curve is the difference pattern, plotted at the same scale as the other patterns. The vertical scale has been multiplied by a factor of 10 for 2θ > 46.0°. The row of black tick marks indicates the reflection positions for this phase.

The initial structural model for NaRbHC_6_H_5_O_7_ was taken from Rammohan & Kaduk (2016 ▸), replacing the K by Rb and changing the lattice parameters to the observed values. Pseudovoigt profile coefficients were as parameterized in Thompson *et al.* (1987 ▸) and the asymmetry correction of Finger *et al.* (1994 ▸) was applied as well as the microstrain broadening description by Stephens (1999 ▸). The hydrogen atoms were included in fixed positions, which were re-calculated during the course of the refinement. Crystal data, data collection and structure refinement (Fig. 7 ▸) details are summarized in Table 3 ▸. The *U*_iso_ of C2, C3, and C4 were constrained to be equal, and those of H7, H8, H9, and H10 were constrained to be 1.3 × that of these carbon atoms. The *U*_iso_ of C1, C5, C6, and the oxygen atoms were constrained to be equal, and that of H18 was constrained to be 1.3 × this value. The *U*_iso_ of H21 and H22 were fixed.

**Figure 8:**
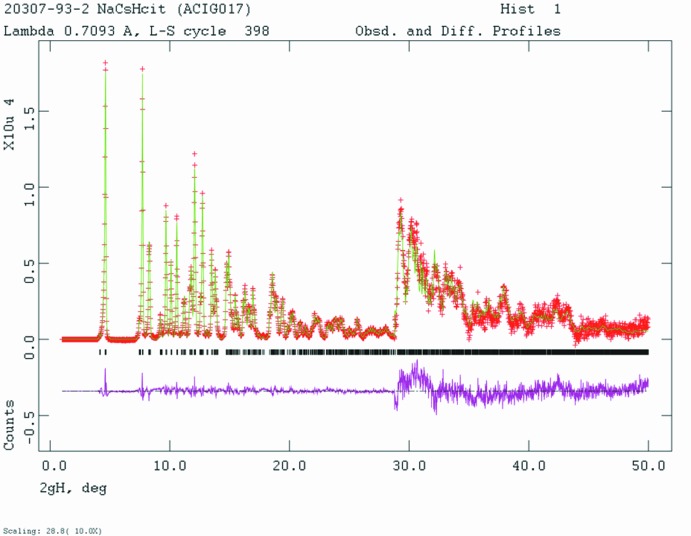
Rietveld plot for NaCsHC_6_H_5_O_7_. The red crosses represent the observed data points, and the green line is the calculated pattern. The magenta curve is the difference pattern, plotted at the same scale as the other patterns. The vertical scale has been multiplied by a factor of 10 for 2θ > 28.8°. The row of black tick marks indicates the reflection positions for this phase.

Analysis of the systematic absences in the pattern of NaCsHC_6_H_5_O_7_ suggested the space groups *I2*, *Im*, or *I2/m*. The volume of the unit cell corresponded to *Z* = 4. Space group *I2* was selected, and confirmed by successful solution and refinement of the structure. The structure was solved with *FOX* (Favre-Nicolin & Černý, 2002 ▸). The maximum sin θ/λ used for structure solution was 0.55 Å, and a citrate, Cs, Na, and O (water mol­ecule) were used as fragments. The solution with the lowest cost factor has the Cs, Na, and O on top of each other, but the Cs was eight-coordinate and all six carboxyl­ate oxygen atoms were coordinated to the Cs atom. The structure was examined for voids using *Materials Studio* (Dassault Systemes, 2017 ▸). One void at approximately 0.375,0.600,0.379 had acceptable coordination to O atoms, and was assigned as Na20. Another void was assigned as O21, but this moved too close to the citrate anion on refinement and was discarded. Active hydrogen atoms were placed by analysis of hydrogen-bonding inter­actions. The refinement strategy (Fig. 8 ▸) was similar to that used for the Rb compound. Cs19 was refined anisotropically.

Density functional geometry optimizations (fixed experimental unit cells) were carried out using *CRYSTAL14* (Dovesi *et al.*, 2014 ▸). The basis sets for the H, C, and O atoms were those of Gatti *et al.* (1994 ▸), the basis sets for Na was that of Dovesi *et al.* (1991 ▸), and the basis sets for Rb and Cs were those of Sophia *et al.* (2014 ▸). The calculations were run on eight 2.1 GHz Xeon cores (each with 6 GB RAM) of a 304-core Dell Linux cluster at Illinois Institute of Technology, using 8 *k*-points and the B3LYP functional, and took 10.8 and 7.5 h.

## Supplementary Material

Crystal structure: contains datablock(s) KADU1716_publ, kadu1716_DFT, ACIG017_publ, acig017_DFT. DOI: 10.1107/S205698901900063X/vn2138sup1.cif

CCDC references: 1890745, 1890746, 1890747, 1890748

Additional supporting information:  crystallographic information; 3D view; checkCIF report

## Figures and Tables

**Figure 2:**
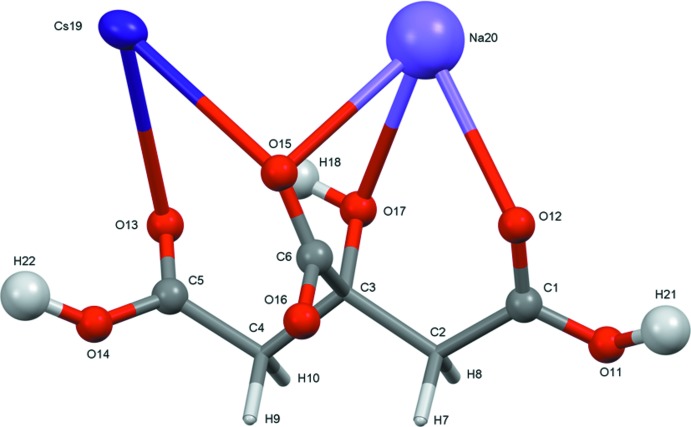
The asymmetric unit of NaCsHC_6_H_5_O_7_, with the atom numbering and 50% probability spheroids.

**Figure 4:**
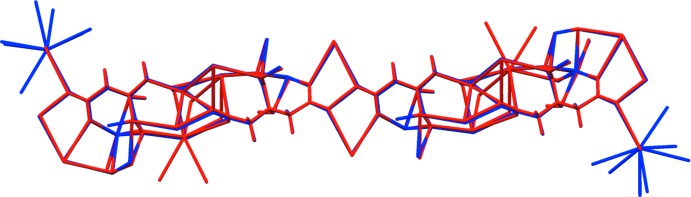
Comparison of the refined and optimized structures of sodium caesium hydrogen citrate. The refined structure is in red, and the DFT-optimized structure is in blue.

**Table 1:** Hydrogen-bond geometry for [NaRb(C_6_H_6_O_7_)]

*D*—H⋯*A*	*D*—H(Å)	H⋯*A*(Å)	*D*⋯*A*(Å)	*D*—H⋯*A*(°)	Mulliken overlap(electrons)	H-bond energy(kcal mol^−1^)
O13—H22⋯O13^i^	1.199	1.199	2.398	180.0	0.143	20.7
O11—H21⋯O11^ii^	1.213	1.213	2.426	180.0	0.140	20.5
O17—H18⋯O15	0.979	1.873	2.575	126.2	0.059	13.3
O17—H18⋯O11^iii^	0.979	2.507	3.180	125.8	0.016	6.9
C2—H8⋯O14^iv^	1.094	2.478	3.541	163.7	0.018	

Symmetry codes: (i) 2 − *x*, 2 − *y*, 2 − *z*; (ii) 1 − *x*, 1 − *y*, 1 − *z*; (iii) 1 + *x*, *y*, *z*; (iv) *x* − 1, *y*, *z*.

**Table 2:** Hydrogen-bond geometry for [NaCs(C_6_H_6_O_7_)]

*D*—H⋯*A*	*D*—H(Å)	H⋯*A*(Å)	*D*⋯*A*(Å)	*D*—H⋯*A*(°)	Mulliken overlap(electrons)	H-bond energy(kcal mol^−1^)
O14—H22⋯O14^i^	1.200	1.200	2.347	156.1	0.133	19.9
O11—H21⋯O11^ii^	1.203	1.203	2.398	170.6	0.143	20.7
O17—H18⋯O13^111^	0.976	1.941	2.779	142.4	0.046	11.7

Symmetry codes: (i) −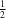 − *x*, −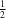 + *y*, 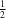 − *z*; (ii) −*x*, *y*, −*z*; (iii) 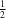 + *x*, −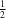 − *y*, −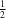 + *z*.

**Table 3:** Experimental details

	[NaRb(C_6_H_6_O_7_)]	[NaCs(C_6_H_6_O_7_)]
Crystal data		
*M*_r_	298.57	346.00
Crystal system, space group	Triclinic, *P* 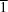	Monoclinic, *I*2
Temperature (K)	300	300
*a*, *b*, *c* (Å)	5.9864 (2), 8.4104 (3), 10.2903 (3)	10.8913 (5), 5.5168 (2), 17.7908 (8)
α, β, γ (°)	74.798 (3), 76.756 (3), 72.878 (2)	90, 97.014 (4), 90
*V* (Å^3^)	471.28 (3)	1060.96 (6)
*Z*	2	4
Radiation type	*K*α_1_, *K*α_2_, λ = 1.540593, 1.544451 Å	*K*α_1_, *K*α_2_, λ = 0.709319, 0.713609 Å
μ (mm^−1^)	–	2.09
Specimen shape, size (mm)	Flat sheet, 24 × 24	Cylinder, 12 × 0.3

Data collection		
Diffractometer	Bruker D2 Phaser	PANalytical Empyrean
Specimen mounting	Standard holder	Glass capillary
Data collection mode	Reflection	Transmission
Scan method	Step	Step
2θ values (°)	2θ_min_ = 5.001 2θ_max_ = 100.007 2θ_step_ = 0.020	2θ_min_ = 1.011 2θ_max_ = 49.991 2θ_step_ = 0.017

Refinement		
*R* factors and goodness of fit	*R*_p_ = 0.028, *R*_wp_ = 0.038, *R*_exp_ = 0.022, *R*(*F* ^2^) = 0.13613, χ^2^ = 3.028	*R*_p_ = 0.045, *R*_wp_ = 0.059, *R*_exp_ = 0.026, *R*(*F* ^2^) = 0.08622, χ^2^ = 5.570
No. of parameters	84	80
No. of restraints	29	29
H-atom treatment	Only H-atom displacement parameters refined	Only H-atom displacement parameters refined

The same symmetry and lattice parameters were used for the DFT calculations as for each powder diffraction study. Computer programs: *DIFFRAC.Measurement* (Bruker, 2009 ▸), *FOX* (Favre-Nicolin & Černý, 2002 ▸), *GSAS* (Larson & Von Dreele, 2004 ▸), *Mercury* (Macrae *et al.*, 2008 ▸), *DIAMOND* (Crystal Impact, 2015 ▸) and *publCIF* (Westrip, 2010 ▸).

## supplementary crystallographic information

### Poly[(µ-hydrogen citrato)rubidiumsodium] (KADU1716_publ) Crystal data

**Table:**

[NaRb(C_6_H_6_O_7_)]	*V* = 471.28 (3) Å^3^
*M**_r_* = 298.57	*Z* = 2
Triclinic, *P*1	*D*_x_ = 2.104 Mg m^−^^3^
Hall symbol: -P 1	Kα_1_, Kα_2_ radiation, λ = 1.540593, 1.544451 Å
*a* = 5.9864 (2) Å	*T* = 300 K
*b* = 8.4104 (3) Å	Particle morphology: powder
*c* = 10.2903 (3) Å	white
α = 74.798 (3)°	flat_sheet, 24 × 24 mm
β = 76.756 (3)°	Specimen preparation: Prepared at 403 K
γ = 72.878 (2)°	

### Poly[(µ-hydrogen citrato)rubidiumsodium] (KADU1716_publ) Data collection

**Table:**

Bruker D2 Phaser diffractometer	Data collection mode: reflection
Radiation source: selaed X-ray tube	Scan method: step
Specimen mounting: standard holder	2θ_min_ = 5.001°, 2θ_max_ = 100.007°, 2θ_step_ = 0.020°

### Poly[(µ-hydrogen citrato)rubidiumsodium] (KADU1716_publ) Refinement

**Table:**

Least-squares matrix: full	84 parameters
*R*_p_ = 0.028	29 restraints
*R*_wp_ = 0.038	2 constraints
*R*_exp_ = 0.022	Only H-atom displacement parameters refined
*R*(*F*^2^) = 0.13613	Weighting scheme based on measured s.u.'s
4701 data points	(Δ/σ)_max_ = 0.03
Profile function: CW Profile function number 4 with 27 terms Pseudovoigt profile coefficients as parameterized in P. Thompson, D.E. Cox & J.B. Hastings (1987). J. Appl. Cryst.,20,79-83. Asymmetry correction of L.W. Finger, D.E. Cox & A. P. Jephcoat (1994). J. Appl. Cryst.,27,892-900. Microstrain broadening by P.W. Stephens, (1999). J. Appl. Cryst.,32,281-289. #1(GU) = 2.580 #2(GV) = 0.000 #3(GW) = 1.999 #4(GP) = 0.000 #5(LX) = 4.181 #6(ptec) = 1.74 #7(trns) = 4.34 #8(shft) = -2.5167 #9(sfec) = 0.00 #10(S/L) = 0.0235 #11(H/L) = 0.0200 #12(eta) = 0.0000 Peak tails are ignored where the intensity is below 0.0050 times the peak Aniso. broadening axis 0.0 0.0 1.0	Background function: GSAS Background function number 1 with 10 terms. Shifted Chebyshev function of 1st kind 1: 1751.95 2: -322.287 3: 62.9433 4: -1.65870 5: 15.3537 6: -30.8122 7: 27.0452 8: -10.7829 9: 5.15006 10: -0.147912

### Poly[(µ-hydrogen citrato)rubidiumsodium] (KADU1716_publ) Fractional atomic coordinates and isotropic or equivalent isotropic displacement parameters (Å^2^)

**Table:**

	*x*	*y*	*z*	*U*_iso_*/*U*_eq_	
C1	0.592 (2)	0.5051 (13)	0.6742 (10)	0.021 (2)*	
C2	0.556 (2)	0.5974 (17)	0.7878 (10)	0.003 (6)*	
C3	0.7655 (17)	0.6762 (10)	0.7701 (7)	0.003 (6)*	
C4	0.744 (2)	0.7373 (16)	0.9020 (8)	0.003 (6)*	
C5	0.905 (3)	0.851 (3)	0.8880 (9)	0.021 (2)*	
C6	0.7512 (17)	0.8313 (11)	0.6487 (8)	0.021 (2)*	
H7	0.54367	0.50387	0.89029	0.004 (7)*	
H8	0.38434	0.70246	0.78480	0.004 (7)*	
H9	0.79156	0.62260	0.98785	0.004 (7)*	
H10	0.55240	0.80973	0.93127	0.004 (7)*	
O11	0.507 (2)	0.5851 (14)	0.5670 (8)	0.021 (2)*	
O12	0.657 (3)	0.3441 (14)	0.6967 (12)	0.021 (2)*	
O13	0.898 (3)	0.9131 (19)	0.9900 (15)	0.021 (2)*	
O14	1.045 (2)	0.8875 (18)	0.7771 (10)	0.021 (2)*	
O15	0.914 (3)	0.8275 (16)	0.5451 (11)	0.021 (2)*	
O16	0.5597 (18)	0.9458 (15)	0.6434 (12)	0.021 (2)*	
O17	0.983 (2)	0.5533 (13)	0.7469 (11)	0.021 (2)*	
H18	1.06896	0.61913	0.65793	0.027 (3)*	
Na19	0.2740 (17)	0.8715 (12)	0.5586 (9)	0.038 (5)*	
Rb20	0.1828 (6)	0.2215 (5)	0.7148 (3)	0.060 (2)*	
H21	0.5	0.5	0.5	0.03*	
H22	1.0	1.0	1.0	0.03*	

### Poly[(µ-hydrogen citrato)rubidiumsodium] (KADU1716_publ) Geometric parameters (Å, º)

**Table:**

C1—C2	1.5091 (17)	O14—Rb20^iv^	3.028 (14)
C1—O11	1.261 (3)	O15—C6	1.269 (3)
C1—O12	1.267 (3)	O15—Na19^ii^	2.332 (16)
C2—C1	1.5091 (17)	O15—Na19^v^	2.504 (13)
C2—C3	1.5403 (17)	O15—Rb20^i^	3.013 (16)
C3—C2	1.5403 (17)	O16—C3	2.429 (6)
C3—C4	1.5392 (17)	O16—C6	1.263 (3)
C3—C6	1.5486 (17)	O16—O15	2.193 (8)
C3—O17	1.419 (3)	O16—O16^v^	3.04 (2)
C4—C3	1.5392 (17)	O16—Na19	2.382 (17)
C4—C5	1.5111 (17)	O16—Na19^v^	2.439 (13)
C5—C4	1.5111 (17)	O16—Rb20^vi^	2.839 (11)
C5—O13	1.275 (3)	O17—C3	1.419 (3)
C5—O14	1.274 (3)	O17—Rb20^ii^	2.769 (10)
C6—C3	1.5486 (17)	Na19—O11	2.393 (16)
C6—O15	1.269 (3)	Na19—O12^i^	3.453 (14)
C6—O16	1.263 (3)	Na19—O14^vii^	2.366 (13)
O11—C1	1.261 (3)	Na19—O15^vii^	2.332 (16)
O11—Na19	2.393 (16)	Na19—O15^v^	2.504 (13)
O11—Rb20^i^	3.366 (12)	Na19—O16	2.382 (17)
O12—C1	1.267 (3)	Na19—O16^v^	2.439 (13)
O12—C2	2.411 (8)	Rb20—O11^i^	3.366 (12)
O12—Rb20	3.246 (14)	Rb20—O12^vii^	3.044 (13)
O12—Rb20^ii^	3.044 (13)	Rb20—O12	3.246 (14)
O13—C5	1.275 (3)	Rb20—O13^iii^	2.931 (16)
O13—Rb20^iii^	2.931 (16)	Rb20—O14^viii^	3.028 (14)
O13—H22	1.117 (10)	Rb20—O15^i^	3.013 (16)
O14—C4	2.433 (14)	Rb20—O16^ix^	2.839 (11)
O14—C5	1.274 (3)	Rb20—O17^vii^	2.769 (10)
O14—Na19^ii^	2.366 (13)		
			
C2—C1—O11	118.7 (8)	O11—Na19—O15^v^	158.0 (5)
C2—C1—O12	120.3 (6)	O11—Na19—O16	92.2 (5)
O11—C1—O12	119.1 (7)	O11—Na19—O16^v^	108.6 (6)
C1—C2—C3	109.8 (5)	O14^vii^—Na19—O15^vii^	75.6 (5)
C2—C3—C4	107.2 (4)	O14^vii^—Na19—O15^v^	93.6 (5)
C2—C3—C6	110.0 (4)	O14^vii^—Na19—O16	84.7 (5)
C2—C3—O17	110.1 (5)	O14^vii^—Na19—O16^v^	140.0 (6)
C4—C3—C6	108.9 (5)	O15^vii^—Na19—O15^v^	82.9 (6)
C4—C3—O17	110.8 (5)	O15^vii^—Na19—O16	159.9 (7)
C6—C3—O17	109.9 (4)	O15^vii^—Na19—O16^v^	114.6 (6)
C3—C4—C5	113.0 (7)	O15^v^—Na19—O16	94.3 (7)
C4—C5—O13	118.6 (6)	O15^v^—Na19—O16^v^	52.7 (2)
C4—C5—O14	121.5 (11)	O16—Na19—O16^v^	78.1 (5)
O13—C5—O14	119.9 (8)	O11^i^—Rb20—O12^vii^	109.5 (3)
C3—C6—O15	119.6 (5)	O11^i^—Rb20—O12	53.0 (3)
C3—C6—O16	119.2 (5)	O11^i^—Rb20—O13^iii^	151.0 (3)
O15—C6—O16	120.1 (6)	O11^i^—Rb20—O14^viii^	130.8 (3)
C1—O11—Na19	118.5 (9)	O11^i^—Rb20—O15^i^	67.2 (3)
C1—O11—Rb20^i^	123.9 (10)	O11^i^—Rb20—O16^ix^	77.9 (3)
Na19—O11—Rb20^i^	81.1 (4)	O11^i^—Rb20—O17^vii^	82.2 (3)
C1—O12—Rb20	105.2 (8)	O12^vii^—Rb20—O12	144.3 (4)
C1—O12—Rb20^ii^	110.5 (10)	O12^vii^—Rb20—O13^iii^	93.8 (4)
Rb20—O12—Rb20^ii^	144.3 (4)	O12^vii^—Rb20—O14^viii^	78.6 (3)
C5—O13—Rb20^iii^	132.3 (10)	O12^vii^—Rb20—O15^i^	68.6 (3)
C5—O14—Na19^ii^	160.1 (13)	O12^vii^—Rb20—O16^ix^	135.6 (4)
C5—O14—Rb20^iv^	118.0 (12)	O12^vii^—Rb20—O17^vii^	66.2 (4)
Na19^ii^—O14—Rb20^iv^	81.6 (5)	O12—Rb20—O13^iii^	98.0 (3)
C6—O15—Na19^ii^	118.8 (12)	O12—Rb20—O14^viii^	137.1 (3)
C6—O15—Na19^v^	90.6 (6)	O12—Rb20—O15^i^	117.4 (3)
C6—O15—Rb20^i^	119.3 (11)	O12—Rb20—O16^ix^	76.0 (3)
Na19^ii^—O15—Na19^v^	97.1 (6)	O12—Rb20—O17^vii^	79.7 (3)
Na19^ii^—O15—Rb20^i^	121.9 (4)	O13^iii^—Rb20—O14^viii^	69.3 (3)
Na19^v^—O15—Rb20^i^	79.8 (4)	O13^iii^—Rb20—O15^i^	140.0 (3)
C6—O16—Na19	112.9 (12)	O13^iii^—Rb20—O16^ix^	97.6 (4)
C6—O16—Na19^v^	93.8 (6)	O13^iii^—Rb20—O17^vii^	92.2 (4)
C6—O16—Rb20^vi^	158.9 (12)	O14^viii^—Rb20—O15^i^	72.0 (3)
Na19—O16—Na19^v^	101.9 (5)	O14^viii^—Rb20—O16^ix^	66.0 (4)
Na19—O16—Rb20^vi^	85.5 (4)	O14^viii^—Rb20—O17^vii^	139.1 (4)
Na19^v^—O16—Rb20^vi^	92.3 (4)	O15^i^—Rb20—O16^ix^	75.4 (4)
O11—Na19—O14^vii^	107.9 (5)	O15^i^—Rb20—O17^vii^	110.9 (4)
O11—Na19—O15^vii^	97.9 (6)	O16^ix^—Rb20—O17^vii^	154.8 (3)

Symmetry codes: (i) −*x*+1, −*y*+1, −*z*+1; (ii) *x*+1, *y*, *z*; (iii) −*x*+1, −*y*+1, −*z*+2; (iv) *x*+1, *y*+1, *z*; (v) −*x*+1, −*y*+2, −*z*+1; (vi) *x*, *y*+1, *z*; (vii) *x*−1, *y*, *z*; (viii) *x*−1, *y*−1, *z*; (ix) *x*, *y*−1, *z*.

### (kadu1716_DFT) Crystal data

**Table:**

C_6_H_6_NaO_7_Rb	α = 74.7995°
*M**_r_* = 298.57	β = 76.7573°
Triclinic, *P*1	γ = 72.8749°
*a* = 5.9859 Å	*V* = 471.23 Å^3^
*b* = 8.4102 Å	*Z* = 2
*c* = 10.2904 Å	

### (kadu1716_DFT) Data collection

**Table:**

*h* = →	*l* = →
*k* = →	

### (kadu1716_DFT) Fractional atomic coordinates and isotropic or equivalent isotropic displacement parameters (Å^2^)

**Table:**

	*x*	*y*	*z*	*U*_iso_*/*U*_eq_	
C1	0.58312	0.48855	0.68564	0.01800*	
C2	0.57651	0.57925	0.79638	0.00600*	
C3	0.76841	0.67872	0.76846	0.00600*	
C4	0.73694	0.75315	0.89436	0.00600*	
C5	0.90081	0.86416	0.88638	0.01800*	
C6	0.74570	0.82427	0.63974	0.01800*	
H7	0.60167	0.48235	0.88975	0.00700*	
H8	0.40172	0.66683	0.81269	0.00700*	
H9	0.76305	0.64923	0.98397	0.00700*	
H10	0.55518	0.83019	0.91496	0.00700*	
O11	0.50420	0.58854	0.57498	0.01800*	
O12	0.65090	0.33239	0.70086	0.01800*	
O13	0.87388	0.91272	0.99960	0.01800*	
O14	1.04257	0.90330	0.78197	0.01800*	
O15	0.91969	0.81409	0.54210	0.01800*	
O16	0.56046	0.94323	0.63928	0.01800*	
O17	0.99694	0.56509	0.74766	0.01800*	
H18	1.06896	0.61913	0.65793	0.02340*	
Na19	0.25929	0.87959	0.56024	0.02900*	
Rb20	0.19358	0.22247	0.71319	0.05030*	
H21	0.50000	0.50000	0.50000	0.03000*	
H22	1.00000	1.00000	1.00000	0.03000*	

### (kadu1716_DFT) Bond lengths (Å)

**Table:**

C1—C2	1.516	C4—H10	1.095
C1—O11	1.318	C5—O13	1.294
C1—O12	1.233	C5—O14	1.243
C2—C3	1.546	C6—O15	1.271
C2—H7	1.092	C6—O16	1.256
C2—H8	1.094	O11—H21	1.213
C3—C4	1.533	O13—H22	1.199
C3—C6	1.551	O17—H18	0.979
C3—O17	1.426	H21—O11^i^	1.213
C4—C5	1.517	H22—O13^ii^	1.199
C4—H9	1.096		

Symmetry codes: (i) −*x*+1, −*y*+1, −*z*+1; (ii) −*x*+2, −*y*+2, −*z*+2.

### (kadu1716_DFT) Hydrogen-bond geometry (Å, º)

**Table:**

*D*—H···*A*	*D*—H	H···*A*	*D*···*A*	*D*—H···*A*
O13—H22···O13	1.199	1.199	2.398	180.0
O11—H21···O11	1.213	1.213	2.426	180.0
O17—H18···O15	0.979	1.873	2.575	126.2
O17—H18···O11	0.979	2.507	3.180	125.8
C2—H8···O14	1.094	2.478	3.541	163.7

### Poly[(µ-hydrogen citrato)caesiumsodium] (ACIG017_publ) Crystal data

**Table:**

[CsNa(C_6_H_6_O_7_)]	*Z* = 4
*M**_r_* = 346.00	*D*_x_ = 2.166 Mg m^−^^3^
Monoclinic, *I*2	Kα_1_, Kα_2_ radiation, λ = 0.709319, 0.713609 Å
Hall symbol: I 2y	µ = 2.09 mm^−^^1^
*a* = 10.8913 (5) Å	*T* = 300 K
*b* = 5.5168 (2) Å	Particle morphology: powder
*c* = 17.7908 (8) Å	white
β = 97.014 (4)°	cylinder, 12 × 0.3 mm
*V* = 1060.96 (6) Å^3^	Specimen preparation: Prepared at 403 K

### Poly[(µ-hydrogen citrato)caesiumsodium] (ACIG017_publ) Data collection

**Table:**

PANalytical Empyrean diffractometer	Data collection mode: transmission
Radiation source: sealed X-ray tube	Scan method: step
Specimen mounting: glass capillary	2θ_min_ = 1.011°, 2θ_max_ = 49.991°, 2θ_step_ = 0.017°

### Poly[(µ-hydrogen citrato)caesiumsodium] (ACIG017_publ) Refinement

**Table:**

Least-squares matrix: full	80 parameters
*R*_p_ = 0.045	29 restraints
*R*_wp_ = 0.059	2 constraints
*R*_exp_ = 0.026	Only H-atom displacement parameters refined
*R*(*F*^2^) = 0.08622	Weighting scheme based on measured s.u.'s
2932 data points	(Δ/σ)_max_ = 0.06
Profile function: CW Profile function number 4 with 21 terms Pseudovoigt profile coefficients as parameterized in P. Thompson, D.E. Cox & J.B. Hastings (1987). J. Appl. Cryst.,20,79-83. Asymmetry correction of L.W. Finger, D.E. Cox & A. P. Jephcoat (1994). J. Appl. Cryst.,27,892-900. Microstrain broadening by P.W. Stephens, (1999). J. Appl. Cryst.,32,281-289. #1(GU) = 53.860 #2(GV) = 0.000 #3(GW) = 0.786 #4(GP) = 0.000 #5(LX) = 1.886 #6(ptec) = 0.00 #7(trns) = 0.00 #8(shft) = 0.0000 #9(sfec) = 0.00 #10(S/L) = 0.0151 #11(H/L) = 0.0173 #12(eta) = 0.5113 #13(S400 ) = 1.1E-01 #14(S040 ) = 4.6E-01 #15(S004 ) = 6.1E-03 #16(S220 ) = 2.3E-01 #17(S202 ) = 3.5E-02 #18(S022 ) = 7.8E-02 #19(S301 ) = 8.2E-02 #20(S103 ) = -1.3E-02 #21(S121 ) = 7.3E-02 Peak tails are ignored where the intensity is below 0.0050 times the peak Aniso. broadening axis 0.0 0.0 1.0	Background function: GSAS Background function number 1 with 3 terms. Shifted Chebyshev function of 1st kind 1: 711.736 2: 51.3623 3: -153.142

### Poly[(µ-hydrogen citrato)caesiumsodium] (ACIG017_publ) Fractional atomic coordinates and isotropic or equivalent isotropic displacement parameters (Å^2^)

**Table:**

	*x*	*y*	*z*	*U*_iso_*/*U*_eq_	
C1	0.5180 (19)	0.183 (7)	0.3879 (6)	0.027 (3)*	
C2	0.5919 (14)	0.118 (3)	0.3242 (6)	0.002 (7)*	
C3	0.5465 (10)	0.234 (2)	0.2470 (5)	0.002 (7)*	
C4	0.6279 (14)	0.138 (3)	0.1886 (6)	0.002 (7)*	
C5	0.588 (2)	0.249 (3)	0.1120 (6)	0.027 (3)*	
C6	0.4103 (11)	0.162 (3)	0.2216 (9)	0.027 (3)*	
H7	0.58724	−0.0875	0.31719	0.003 (9)*	
H8	0.69162	0.17452	0.33886	0.003 (9)*	
H9	0.61841	−0.06615	0.18447	0.003 (9)*	
H10	0.72772	0.19018	0.20703	0.003 (9)*	
O11	0.5625 (17)	0.129 (4)	0.4554 (6)	0.027 (3)*	
O12	0.4121 (17)	0.285 (4)	0.3756 (9)	0.027 (3)*	
O13	0.601 (3)	0.476 (3)	0.1023 (8)	0.027 (3)*	
O14	0.5515 (19)	0.112 (3)	0.0558 (7)	0.027 (3)*	
O15	0.3392 (15)	0.319 (3)	0.1871 (10)	0.027 (3)*	
O16	0.3821 (15)	−0.062 (3)	0.2172 (12)	0.027 (3)*	
O17	0.5558 (14)	0.490 (2)	0.2509 (8)	0.027 (3)*	
H18	0.54799	0.56496	0.20157	0.036 (4)*	
Cs19	0.3269 (3)	0.70766	0.05362 (15)	0.04276	
Na20	0.3483 (18)	0.742 (6)	0.2891 (9)	0.124 (8)*	
H21	0.5	0.102	0.5	0.05*	
H22	0.5	0.124	0.0	0.05*	

### Poly[(µ-hydrogen citrato)caesiumsodium] (ACIG017_publ) Atomic displacement parameters (Å^2^)

**Table:**

	*U*^11^	*U*^22^	*U*^33^	*U*^12^	*U*^13^	*U*^23^
Cs19	0.039 (3)	0.038 (3)	0.051 (3)	0.006 (5)	0.006 (2)	−0.007 (5)

### Poly[(µ-hydrogen citrato)caesiumsodium] (ACIG017_publ) Geometric parameters (Å, º)

**Table:**

C1—C2	1.512 (2)	O14—Cs19^iv^	3.341 (19)
C1—O11	1.274 (7)	O15—C6	1.272 (7)
C1—O12	1.278 (7)	O15—Cs19	3.189 (17)
C2—C1	1.512 (2)	O15—Na20	2.95 (3)
C2—C3	1.540 (2)	O15—Na20^i^	2.18 (2)
C3—C2	1.540 (2)	O16—C6	1.272 (7)
C3—C4	1.540 (2)	O16—Cs19^iii^	3.17 (2)
C3—C6	1.548 (2)	O16—Na20^iii^	1.75 (3)
C3—O17	1.419 (7)	O16—Na20^i^	3.01 (3)
C4—C3	1.540 (2)	O17—C3	1.419 (7)
C4—C5	1.509 (2)	O17—Na20	2.81 (3)
C5—C4	1.509 (2)	Cs19—O11^v^	3.211 (17)
C5—O13	1.272 (7)	Cs19—O12^vi^	3.057 (15)
C5—O14	1.275 (7)	Cs19—O13	3.27 (3)
C6—C3	1.548 (2)	Cs19—O13^ii^	3.236 (17)
C6—O15	1.272 (7)	Cs19—O14^vii^	3.309 (17)
C6—O16	1.272 (7)	Cs19—O14^viii^	3.341 (19)
O11—C1	1.274 (7)	Cs19—O15	3.189 (17)
O12—C1	1.278 (7)	Cs19—O16^vii^	3.17 (2)
O12—Cs19^i^	3.057 (15)	Cs19—H18	3.435 (3)
O12—Na20	2.99 (4)	Na20—O12	2.99 (4)
O13—C5	1.272 (7)	Na20—O15	2.95 (3)
O13—Cs19	3.27 (3)	Na20—O15^vi^	2.18 (2)
O13—Cs19^ii^	3.236 (17)	Na20—O16^vii^	1.75 (3)
O14—C5	1.275 (7)	Na20—O16^vi^	3.01 (3)
O14—O14^ii^	2.16 (3)	Na20—O17	2.81 (3)
O14—Cs19^iii^	3.309 (17)		
			
C2—C1—O11	118.3 (5)	C3—O17—H18	112.6 (12)
C2—C1—O12	121.9 (7)	O11^v^—Cs19—O12^vi^	59.4 (3)
O11—C1—O12	119.8 (6)	O11^v^—Cs19—O13	145.2 (5)
C1—C2—C3	115.3 (5)	O11^v^—Cs19—O13^ii^	76.9 (5)
C2—C3—C4	108.1 (6)	O11^v^—Cs19—O14^vii^	135.3 (5)
C2—C3—C6	110.2 (6)	O11^v^—Cs19—O14^viii^	99.6 (4)
C2—C3—O17	110.9 (6)	O11^v^—Cs19—O15	105.5 (4)
C4—C3—C6	108.9 (6)	O11^v^—Cs19—O16^vii^	127.7 (5)
C4—C3—O17	109.3 (6)	O12^vi^—Cs19—O13	137.9 (6)
C6—C3—O17	109.4 (6)	O12^vi^—Cs19—O13^ii^	135.7 (6)
C3—C4—C5	110.0 (6)	O12^vi^—Cs19—O14^vii^	124.5 (5)
C4—C5—O13	119.6 (8)	O12^vi^—Cs19—O14^viii^	124.4 (5)
C4—C5—O14	119.7 (6)	O12^vi^—Cs19—O15	75.3 (5)
O13—C5—O14	120.4 (7)	O12^vi^—Cs19—O16^vii^	68.9 (5)
C3—C6—O15	118.0 (6)	O13—Cs19—O13^ii^	76.3 (5)
C3—C6—O16	118.9 (7)	O13—Cs19—O14^vii^	67.1 (4)
O15—C6—O16	120.4 (6)	O13—Cs19—O14^viii^	90.1 (5)
C1—O11—Cs19^ix^	134 (2)	O13—Cs19—O15	65.5 (4)
C1—O12—Cs19^i^	131.6 (15)	O13—Cs19—O16^vii^	81.3 (4)
C5—O13—Cs19	107.5 (19)	O13^ii^—Cs19—O14^vii^	91.2 (5)
C5—O13—Cs19^ii^	123.3 (10)	O13^ii^—Cs19—O14^viii^	67.1 (3)
Cs19—O13—Cs19^ii^	85.8 (4)	O13^ii^—Cs19—O15	112.4 (5)
C5—O14—Cs19^iii^	124.4 (14)	O13^ii^—Cs19—O16^vii^	155.3 (6)
C5—O14—Cs19^iv^	138.3 (18)	O14^vii^—Cs19—O14^viii^	37.9 (4)
Cs19^iii^—O14—Cs19^iv^	83.5 (4)	O14^vii^—Cs19—O15	118.7 (4)
C6—O15—Cs19	141.8 (14)	O14^vii^—Cs19—O16^vii^	70.2 (4)
C6—O15—Na20^i^	107.7 (14)	O14^viii^—Cs19—O15	154.1 (4)
Cs19—O15—Na20^i^	108.7 (8)	O14^viii^—Cs19—O16^vii^	102.9 (4)
C6—O16—Cs19^iii^	117.6 (13)	O15—Cs19—O16^vii^	66.3 (3)
C6—O16—Na20^iii^	129 (2)	O15^vi^—Na20—O16^vii^	107.9 (15)
Cs19^iii^—O16—Na20^iii^	113.1 (11)		

Symmetry codes: (i) −*x*+1/2, *y*−1/2, −*z*+1/2; (ii) −*x*+1, *y*, −*z*; (iii) *x*, *y*−1, *z*; (iv) −*x*+1, *y*−1, −*z*; (v) *x*−1/2, *y*+1/2, *z*−1/2; (vi) −*x*+1/2, *y*+1/2, −*z*+1/2; (vii) *x*, *y*+1, *z*; (viii) −*x*+1, *y*+1, −*z*; (ix) *x*+1/2, *y*−1/2, *z*+1/2.

### (acig017_DFT) Crystal data

**Table:**

C6H6CsNaO_7_	*c* = 17.7909 Å
*M**_r_* = 346.0	β = 97.0160°
Monoclinic, *I*2	*V* = 1060.98 Å^3^
*a* = 10.8918 Å	*Z* = 4
*b* = 5.5166 Å	

### (acig017_DFT) Data collection

**Table:**

DFT calculation	*k* = →
*h* = →	*l* = →

### (acig017_DFT) Fractional atomic coordinates and isotropic or equivalent isotropic displacement parameters (Å^2^)

**Table:**

	*x*	*y*	*z*	*U*_iso_*/*U*_eq_	
C1	0.01539	−0.29133	−0.11491	0.02190*	
C2	0.08720	−0.37073	−0.17866	0.00770*	
C3	0.04485	−0.25744	−0.25560	0.00770*	
C4	−0.36770	0.15585	0.18731	0.00770*	
C5	−0.40727	0.26100	0.10948	0.02190*	
C6	−0.09031	−0.33701	−0.28568	0.02190*	
H7	0.07745	0.43212	−0.18341	0.01000*	
H8	0.18550	−0.33438	−0.16183	0.01000*	
H9	−0.36925	−0.04136	0.18368	0.01000*	
H10	−0.27329	0.21290	0.20759	0.01000*	
O11	0.06648	−0.36068	−0.04898	0.02190*	
O12	−0.08435	−0.17967	−0.12642	0.02190*	
O13	−0.41832	0.48425	0.09987	0.02190*	
O14	−0.43234	0.10103	0.05664	0.02190*	
O15	0.33500	0.32450	0.18636	0.02190*	
O16	0.38339	−0.05889	0.21767	0.02190*	
O17	0.05257	−0.00031	−0.24718	0.02190*	
H18	0.04799	0.06496	−0.29843	0.02800*	
Cs19	0.31925	−0.29064	0.05016	0.04080*	
Na20	−0.15569	0.10811	−0.21537	0.10800*	
H21	0.00000	−0.34288	0.00000	0.05000*	
H22	0.00000	−0.35387	0.50000	0.05000*	

### (acig017_DFT) Bond lengths (Å)

**Table:**

C1—C2	1.519	O15—Na20^xii^	2.339
C1—O11	1.293	O16—C6^xii^	1.260
C1—O12	1.244	O16—Cs19	3.240
C2—C3	1.524	O16—Na20^xiii^	2.258
C2—H7^i^	1.095	O16—Na20^vii^	2.641
C2—H8	1.095	O17—H18	0.976
C3—C4^ii^	1.551	O17—Na20	2.477
C3—C6	1.566	Cs19—Cs19^vi^	5.517
C3—O17	1.428	Cs19—Cs19^i^	5.517
C4—C3^iii^	1.551	Cs19—O15^i^	3.210
C4—C5	1.515	Cs19—O12^vii^	3.100
C4—H9	1.090	Cs19—O13^xiv^	3.143
C4—H10	1.094	Cs19—O14^xv^	3.453
C5—O13	1.247	Cs19—O14^vii^	3.220
C5—O14	1.294	Cs19—O13^xvi^	3.246
C6—O15^iv^	1.267	Cs19—Cs19^xvii^	4.517
C6—O16^iv^	1.260	Cs19—Na20^xiii^	4.184
C6—Na20^v^	2.785	Cs19—Na20^vii^	4.234
H7—C2^vi^	1.095	Na20—O15^vii^	2.398
O11—Cs19	3.107	Na20—C6^xviii^	2.785
O11—H21	1.203	Na20—Na20^xviii^	3.568
O12—Cs19^vii^	3.100	Na20—Na20^v^	3.568
O12—Na20	2.308	Na20—O16^xi^	2.258
O13—Cs19^viii^	3.143	Na20—O15^iv^	2.339
O13—Cs19^ix^	3.246	Na20—O16^vii^	2.641
O14—Cs19^x^	3.453	Na20—Cs19^xi^	4.184
O14—Cs19^vii^	3.220	Na20—Cs19^vii^	4.234
O14—H22^xi^	1.200	H21—O11^vii^	1.203
O15—C6^xii^	1.267	H22—O14^xix^	1.200
O15—Cs19^vi^	3.210	H22—O14^xiii^	1.200
O15—Na20^vii^	2.398		

Symmetry codes: (i) *x*, *y*−1, *z*; (ii) *x*+1/2, *y*−1/2, *z*−1/2; (iii) *x*−1/2, *y*+1/2, *z*+1/2; (iv) *x*−1/2, *y*−1/2, *z*−1/2; (v) −*x*−1/2, *y*−1/2, −*z*−1/2; (vi) *x*, *y*+1, *z*; (vii) −*x*, *y*, −*z*; (viii) *x*−1, *y*+1, *z*; (ix) −*x*, *y*+1, −*z*; (x) *x*−1, *y*, *z*; (xi) *x*−1/2, *y*+1/2, *z*−1/2; (xii) *x*+1/2, *y*+1/2, *z*+1/2; (xiii) *x*+1/2, *y*−1/2, *z*+1/2; (xiv) *x*+1, *y*−1, *z*; (xv) *x*+1, *y*, *z*; (xvi) −*x*, *y*−1, −*z*; (xvii) −*x*+1, *y*, −*z*; (xviii) −*x*−1/2, *y*+1/2, −*z*−1/2; (xix) −*x*−1/2, *y*−1/2, −*z*+1/2.

### (acig017_DFT) Hydrogen-bond geometry (Å, º)

**Table:**

*D*—H···*A*	*D*—H	H···*A*	*D*···*A*	*D*—H···*A*
O14—H22···O14	1.200	1.200	2.347	156.1
O11—H21···O11	1.203	1.203	2.398	170.6
O17—H18···O13	0.976	1.941	2.779	142.4
